# Supplementary material for: Reprogramming mRNA Expression in Response to Defect in RNA Polymerase III Assembly in the Yeast Saccharomyces cerevisiae
Source: Int J Mol Sci. 2021 Jul 7;22(14):7298. doi: 10.3390/ijms22147298 (PMC8306304; doi:10.3390/ijms22147298)
Supplement: Supplementary file 1 [file ijms-22-07298-s001.zip › Legends to Supplementary Figures and Tables.pdf]

Legends to Supplementary Figures and Tables

Figure S1. *RBS1* allele is dominant over the *RBS1* R3H allele. A genetic analysis was performed, based on the suppression of cold sensitivity of the *rpc128-1007* mutant. When both *RBS1* and *RBS1*-R3H alleles were simultaneously expressed in the same cells (*rpc128-1007* or *rbs1*Δ *rpc128-1007*), suppression was observed (strain 5 in both panels). This means that the *RBS1*-R3H allele, which does not provide suppression by itself (strains 4 and 6, lower panel) is recessive.

Figure S2. RNA-seq analysis. (**A**) Hierarchical clustermap of differences between RNA-seq data and a good level of reproducibility. The Euclidean distance between each sample was calculated and plotted on heatmap. Data were clustered and presented as a tree (generated by *pheatmap* package in R) on the left side of heatmap. (**B**) Table presenting GO therm analysis of differentially expressed genes. Two functional categories with the lowest p-values were presented for each cluster. (**C)** Boxplots of changes in mRNA levels for genes involved in ribosome biogenesis factors, and mitochondrial translation. Statistical significance was calculated using the Mann-Whitney test. ***, ** and * asterisks indicate, respectively, *p*-value <0.05, <0.01 and <0.001.Asterisks just under boxes present *p*-value of comparison to level in wt, other comparisons are annotated by lines.

Figure S3. RNA-seq results for selected genes validated by RT-PCR. RNAs that were isolated from the *rpc128-1007* mutant that was transformed with the indicated plasmids and control strain (wt) were analyzed by RT-qPCR with specific probes. Bars represent the mean ± standard deviation of three independent experiments. Values of *p* were calculated using a two-tailed *t*-test. ***, ** and * asterisks indicate, respectively, *p*-value <0.001, <0.01 and <0.05. Asterisks just under bars present p-value of comparison to level in wt, other comparisons are annotated by lines.

Table S1. Oligonucleotides for RT-qPCR

Table S2. Summary of RNA-seq data obtained from *DESeq2* quantification. Yeast genes are ordered according to their chromosomal locations. Standard and common names of the respective open reading frames were specified in columns A and B, whereas the column C contains their short descriptions taken from SGD database (www.yeastgenome.org). *rpc128-1007* mutant harboring native *RPC128* gene on the centromeric plasmid pSA23-RET1 was used as wild type control and referred as wt. The numbers in columns D-J represent fold change values of the respective reads, whereas adjusted *p*-values for each comparison were shown in columns L-Q. NA description in fold change appears when there is no counts for the respective gene. NA values for *p*-adjustment mean the presence of outlier counts for gene or low mean normalized counts, what correspond to low quality or nonreplicative data for those genes. For details check http://bioconductor.org/packages/devel/bioc/vignettes/DESeq2/inst/doc/DESeq2.html Gcn4 genes (according to[17,18] are specified in column R.

Genes were designated as differentially expressed when *p*-adjustment is below 0.05. Differentially expressed genes were marked in yellow.
