# Supplementary material for: Reprogramming mRNA Expression in Response to Defect in RNA Polymerase III Assembly in the Yeast Saccharomyces cerevisiae
Source: Int J Mol Sci. 2021 Jul 7;22(14):7298. doi: 10.3390/ijms22147298 (PMC8306304; doi:10.3390/ijms22147298)
Supplement: Supplementary file 1 [file ijms-22-07298-s001.zip › Table S1.pdf]

| Gene | Forward | Reverse |
| --- | --- | --- |
| *ACT1* | CATGTTCCCAGGTATTGCCGA | GTCAAAGAAGCCAAGATAGA |
| *ARG1* | CAGATACCACCCCACCAAAG | TGACTGGAAGACCACGTTCA |
| *ARG3* | TGTATTTTTGCCCGTGTGAA | TCACAAATTGCTTGCAAAGG |
| *BAT1* | TCTTGACCCATCTGCTTGTG | TACGGGCCATGTTCTTATCC |
| *HXT7* | AGCCTCCCACTCGGTTTTAT | AACGACAGGTTCGTGCTCTT |
| *HXT10* | CGGAATCGCCCAGATATTTA | CTCAAAAACAACGCCTGGAT |
| *OAC1* | GCCGCATCTGGTATAATTGG | CGGTGTAGTGCGTTTGTTCA |
