# Supplementary figures and images for: Reprogramming mRNA Expression in Response to Defect in RNA Polymerase III Assembly in the Yeast Saccharomyces cerevisiae
Source: Int J Mol Sci. 2021 Jul 7;22(14):7298. doi: 10.3390/ijms22147298 (PMC8306304; doi:10.3390/ijms22147298)

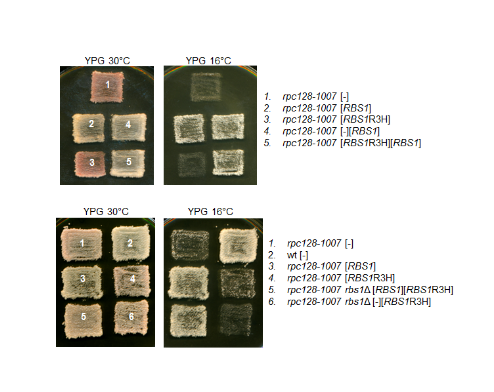

Supplement: Supplementary file 1 [file ijms-22-07298-s001.zip › Fig S1.tif]

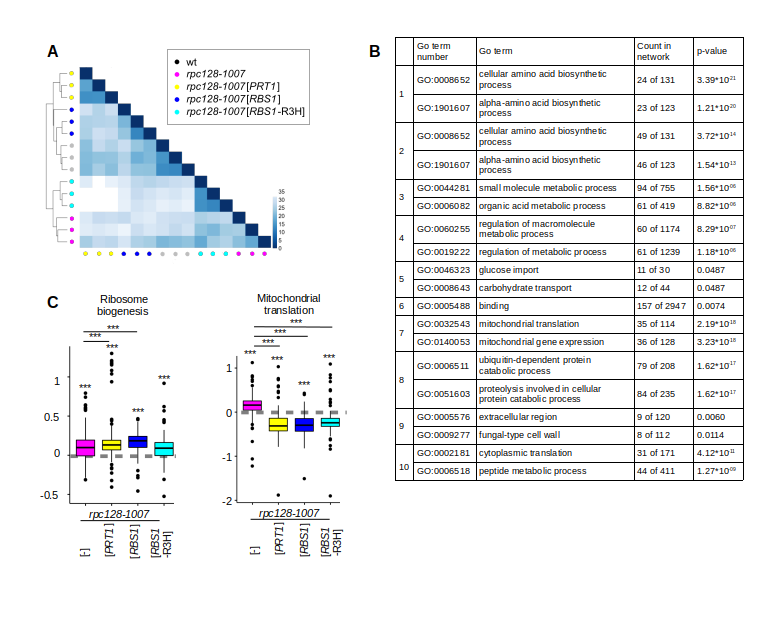

Supplement: Supplementary file 1 [file ijms-22-07298-s001.zip › Fig S2.tif]

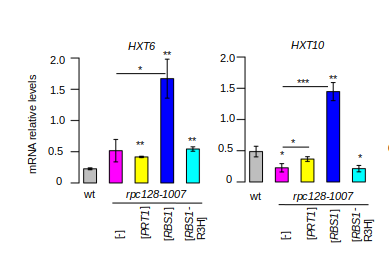

Supplement: Supplementary file 1 [file ijms-22-07298-s001.zip › Fig S3.tif]
